# Preliminary results of “Choose Life!” - a county-wide programme for suicide prevention and mental health awareness

**DOI:** 10.1192/j.eurpsy.2024.519

**Published:** 2024-08-27

**Authors:** R. Wernigg

**Affiliations:** Department for Primary Care Planning and Development, National Directorate-General for Hospitals, Budapest, Hungary

## Abstract

**Introduction:**

Suicidality and depression awareness still remains a concern in Hungary. This programme, based on the principles of the European Union Against Depression, implemented its five steps, such as: 1. improving family doctors’ readiness to diagnose and treat depression, 2. increasing public awareness, 3. training stakeholders and community facilitators, 4. offering special help for risk groups, 5. facilitating self-help by the online tool “ifightdepression” in five of seven districts of Heves county from November 2014 until May 2016.

**Objectives:**

We aimed to look at some clinical outcome measures of the programme, like diagnosis density of depression in primary care before and after the intervention; diagnosis density of depression in outpatient services; suicide attempts in specialised care; and completed suicide rates.

**Methods:**

We extracted patient turnover data from the joint database of the National Healthcare Fund and the National Directorate-General for Hospitals. Raw patient turnover data were divided by the total patient turnover in order to obtain diagnosis density. For the diagnosis of depression, we used the sum of the ICD-10 diagnoses of F32 (depressive episode) to F33 (recurrent depression) plus F4120 (mixed anxiety-depressive disorder), as family doctors tend to use these diagnoses interchangeably. For suicide attempts, we used the diagnoses X60 to X84, plus Y8700. For completed suicides we used the same diagnoses with the “deceased” flag. Diagnosis densities were compared with concurrent national data and were standardised to the long-term average. In the case of outpatient services, we only could retrieve monthly data, which we smoothed out with threemonthly moving averages.

**Results:**

Baseline diagnosis density of depression in primary care was already 44% above the national average when the program started and after the kickoff, it shortly went up to 53,3% and remained over the baseline for as long as until 2019. Also, the recognition rates of depression with no comorbidities in primary care increased by 6%, and steadily remained over the national average until 2019. For outpatient psychiatry, there was an 8% increase in depression turnover throughout the duration of the programme. As regards to suicide attempts treated in hospital, the rates went 20% below the national averages for the duration of the programme, and mostly remained there until 2020. Fatal suicidal events accounted for five to seven deaths a year per county, therefore, simple statistical methods could not uncover significant differences.

**Conclusions:**

These early results indicate that the programme may have been effective in terms of reinforcing the diagnostic and treatment capacities of primary care for recognising a treating depression adequately, thereby eliminating suicide risk. Further statistical exploration of the data is still needed to confirm the magnitude and the validity of these results.

**Disclosure of Interest:**

None Declared

